# Impact of Proximal Conjoint Tendon Injury on Return to Play in the BF–ST Complex: A Prospective MRI-Based Study

**DOI:** 10.3390/diagnostics16010166

**Published:** 2026-01-05

**Authors:** Makoto Wada, Takumi Okunuki, Takeshi Sugimoto, Yasuhito Tanaka, Tsukasa Kumai

**Affiliations:** 1 Department of Orthopedic Surgery, Wada Orthopedic Clinic, Osaka 573-0163, Japan; 2 Waseda Institute for Sport Sciences, Saitama 359-1192, Japan; 3 Graduate School of Sport Sciences, Waseda University, Saitama 359-1192, Japan; 4 Research Organization of Science and Technology, Ritsumeikan University, Shiga 525-8577, Japan; 5 Department of Orthopedic Surgery, Osaka Global Orthopedic Hospital, Osaka 536-0008, Japan; 6 Department of Orthopedic Surgery, Nara Medical University, Nara 634-8521, Japan; 7 Faculty of Sport Sciences, Waseda University, Saitama 359-1192, Japan

**Keywords:** hamstring injury, conjoint tendon, biceps femoris–semitendinosus complex (BT-ST complex), musculotendinous junction (MTJ), intratendinous injury, return to play (RTP), magnetic resonance imaging (MRI), athlete rehabilitation

## Abstract

**Background/Objectives:** Proximal hamstring injuries involving the biceps femoris–semitendinosus (BF–ST) conjoint tendon (CT) often exhibit delayed healing, yet the prognostic significance of CT involvement and intratendinous injury morphology has not been fully clarified. This study aimed to determine whether full-layer CT injury, particularly bilateral involvement in Zone C, prolongs return-to-play (RTP) in competitive rugby athletes. **Methods:** This prospective study evaluated 41 university rugby players with acute BF–ST complex injuries using clinical examination, ultrasonography, and MRI. Injuries were classified by Type (I: full-layer CT; II: BFLH-only; III: ST-only), Zone (A–E), and Grade (0–3). RTP was defined as unrestricted return to team training or match play. Group differences were analyzed using ANOVA or non-parametric tests with appropriate post hoc corrections. **Results:** Type I injuries required significantly longer RTP (11.4 ± 4.8 weeks) than Type II (5.3 ± 2.4 weeks) and Type III (4.0 ± 1.7 weeks), confirming the strong impact of CT involvement on prognosis. In Zone C, bilateral full-layer CT involvement was associated with an approximately twofold longer RTP duration compared with unilateral BFLH-side injuries, indicating that intratendinous tissue disruption influences recovery. These findings highlight the importance of early MRI-based assessment to identify clinically relevant tendon involvement patterns. **Conclusions:** Full-layer CT injuries, particularly bilateral intratendinous patterns in Zone C, markedly prolong RTP compared with isolated BFLH or ST injuries. An MRI-based classification incorporating injury type, zone, and extent of CT involvement provides clinically valuable prognostic information and may enhance RTP decision-making.

## 1. Introduction

Hamstring injuries are a frequent and persistent problem in competitive sports and have long been recognized as a major cause of time loss [1,2]. Epidemiological studies have shown a continuous increase, with professional football demonstrating a yearly rise in incidence and hamstring strains now accounting for nearly one-quarter of all injuries [3,4]. Although eccentric strengthening can reduce occurrence [5] and the mechanisms and risk factors have been increasingly clarified, hamstring injuries remain a significant clinical and performance challenge [6,7,8,9,10].

Among hamstring muscle tears, the ST-BF complex is drawing attention because injuries spanning from the origin at the ischial tuberosity to the musculotendinous junction are frequent and require a long recovery time for return to play (RTP) [11,12,13,14,15]. Anatomically, the semitendinosus (ST) and BFLH share a common origin at the ischial tuberosity, forming the conjoint tendon (CT), whereas the semimembranosus (SM) arises separately [16,17,18,19,20]. The CT represents a structurally unique tendinous unit bridging the medial and lateral hamstrings and transmits tractional forces from both muscles during hip extension and knee flexion.

Recent biomechanical and electromyographic studies have highlighted the unique loading environment of this region. BFLH activation increases markedly during the acceleration phase of sprinting, whereas ST activation increases at top speed, demonstrating phase-dependent functional differences between the two muscles [21]. Furthermore, during the terminal swing phase and initial ground contact, the BFLH generates an external-rotation moment while the ST generates an internal-rotation moment, creating opposing tensile forces that converge on the CT [22]. Muscle functional MRI studies have also shown that hip extension relies more on the BFLH and SM than on the ST, supporting the biomechanical vulnerability of the CT during high-speed movements [23]. These opposing loading patterns suggest that when the CT is injured, realignment of tendon fibers during healing may be disrupted, predisposing the region to delayed recovery and re-injury.

Previous imaging and anatomical studies have further described the structural vulnerability of the CT region. Forlizzi et al. stated that the biceps femoris tendon and semitendinosus tendon fuse to form the common tendon and investigated the severity classification of CT injuries on MRI, as well as return to play (RTP) and patient-reported treatment satisfaction [24]. Rubin et al. [25] reported that avulsion injuries from the ischial tuberosity most frequently involve the conjoint tendon rather than the biceps femoris alone. Azzopardi et al. [26] demonstrated that the angle of the CT’s origin may predispose it to increased strain. These anatomical observations support the hypothesis that CT injuries represent a distinct clinical entity [24,25,26,27,28]. We previously described the ultrasound morphology of the proximal hamstring originate tendon [29], providing preliminary structural insights relevant to proximal hamstring injuries. However, despite growing recognition of the CT’s structural and biomechanical role, few clinical studies have specifically examined the prognostic impact of CT involvement on recovery time. Although past classifications, such as the Munich Consensus [30,31] and the BAMIC system [32], have helped standardize the description of hamstring injury morphology, none distinguish CT injury as a unique pathological category. The prognostic relevance of injury location—tendon origin (Zone A), free tendon (Zone B), and the proximal musculotendinous junction (Zone C)—also remains insufficiently understood. RTP also remains unclear when comparing full-thickness injuries at the musculotendinous junction (Zone C) with unilateral injuries affecting either the BFLH or the CT, where the latter are not full-thickness.

To date, no study has directly compared recovery times for ST injuries without CT involvement, BFLH injuries without CT involvement, and full-layer CT injuries involving both muscles. We hypothesized that injuries involving the ST–BFLH conjoint tendon are associated with longer RTP compared with injuries limited to either the BFLH or ST alone. We also hypothesized that differences in recovery time between unilateral and bilateral injuries would vary at the musculotendinous junction of the CT. Therefore, we performed detailed MRI-based classification of proximal hamstring injuries in university rugby athletes by Type (conjoint tendon vs. BFLH-only vs. ST-only), Zone (A–E), and Grade (0–3). Regarding CT injuries, we investigated differences in recovery times between the ischial tuberosity and the musculotendinous junction, and within the musculotendinous junction, whether the injury was a full-thickness CT injury, bilateral BFLH, or unilateral ST injury.

## 2. Methods

### 2.1. Participants

This prospective study involved male university rugby league players who presented to our clinic with posterior thigh pain initially suspected to be hamstring strains. The study period extended from September 2020 to April 2025. After detailed medical history taking, players with no prior hamstring strain in the same region were included.

Individuals with a previous hamstring muscle strain in a different region were included if more than one year had elapsed since full recovery. Athletes with a history of muscle strain in the contralateral leg or treatment for other injuries were also included provided they had achieved complete recovery.

**Figure 1:**
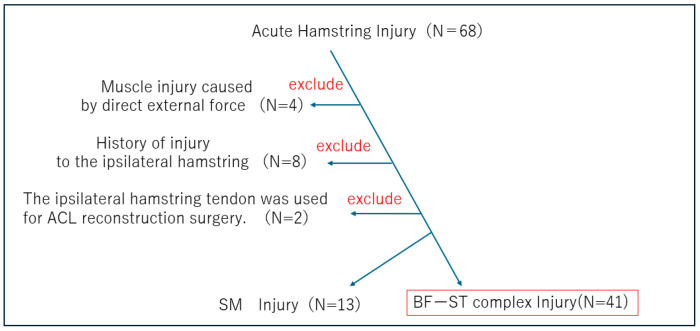
Flow diagram of participant selection according to the STROBE guidelines.

Exclusion criteria comprised cases in which a direct external force may have been applied and individuals with a history of anterior cruciate ligament (ACL) reconstruction using hamstring tendon grafts. Participant inclusion and exclusion, from initial assessment to the final study population, are summarized in a flow diagram in accordance with the STROBE guidelines (Figure 1).

### 2.2. Diagnosis

Diagnosis was based on physical examination, ultrasonography (US), and MRI evaluation. The diagnostic procedure was as follows:

Physical examination: visual inspection, palpation, assessment of joint range of motion, identification of the site of pain by resisted muscle contraction, and detection of extension pain using the Straight Leg Raise test. Ultrasonography: US was performed primarily at the site of pain using a short-axis technique, followed by long-axis evaluation.

MRI: All athletes suspected of having a hamstring muscle tear based on physical examination and ultrasound underwent MRI. MRI examinations were performed using a 3.0-T MR imaging unit (Discovery MR750W; GE Healthcare Technologies, Inc., Chicago, IL, USA). All examinations were conducted using the same field strength. Imaging was obtained in the axial, coronal and sagittal planes. The MRI protocol included T1-weighted sequences for anatomical reference and fat-suppressed T2-weighted sequences, which were used as the primary sequences for diagnostic evaluation. Slice thickness ranged from 5 to 7 mm, which was considered appropriate for musculoskeletal assessment of the proximal hamstring region. Diagnostic evaluation focused on signal intensity changes at the musculotendinous junction and the proximal hamstring conjoint tendon on fat-suppressed T2-weighted images. The final diagnosis was confirmed by two physicians with more than 20 years of experience.

Inclusion of SM injuries: Cases involving isolated semimembranosus (SM) injury identified by MRI were included solely for frequency analysis. The present study on return-to-play (RTP) timing focused exclusively on injuries affecting the BF–ST complex (BFLH, BFSH, ST).

### 2.3. Classification of Injury Site Type/Zone and Severity in the BF–ST Complex

**Figure 2:**
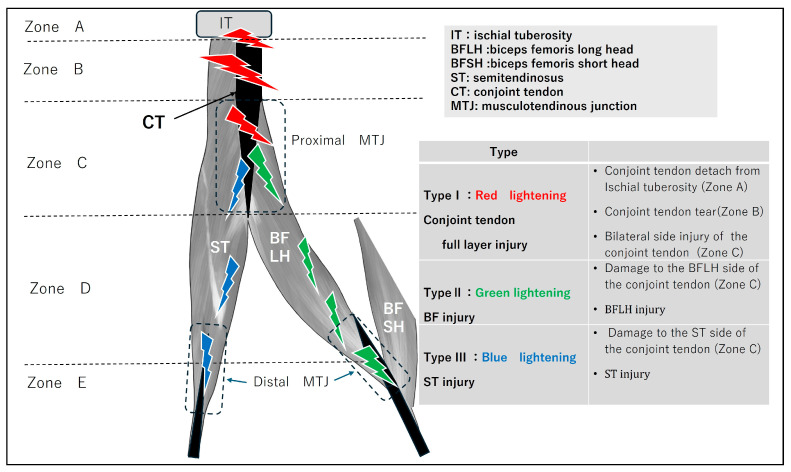
Type classification of BF–ST complex injury. Injury Site (Zone) A: Osteotendinous junction Injury involving detachment of the conjoint tendon from the ischial tuberosity. B: Conjoint tendon Injury from the ischial tuberosity extending to just before the start of the BFLH musculotendinous junction. C: Proximal musculotendinous junction Injury to the musculotendinous junction of the conjoint tendon: BFLH-side, ST-side, or both. D: Intermuscular and myofascial junction Injury at the intermuscular or myofascial junction between BF and ST muscles. E: Distal musculotendinous junction and distal tendon Injury at the distal musculotendinous junction or distal tendon of BF or ST. Severity (Criticality) Classification Grade 0: Pain and physical findings present but no abnormalities detected on MRI. Grade 1: Mild injury with a small hematoma or a limited tear. Grade 2: Moderate injury with a short discontinuity of the injured tendon and mild tendon waviness or distal tearing. Grade 3: Severe injury characterized by marked tendon tortuosity resulting from loss of tension due to rupture, large discontinuity surfaces, and significant retraction.

**Figure 3:**
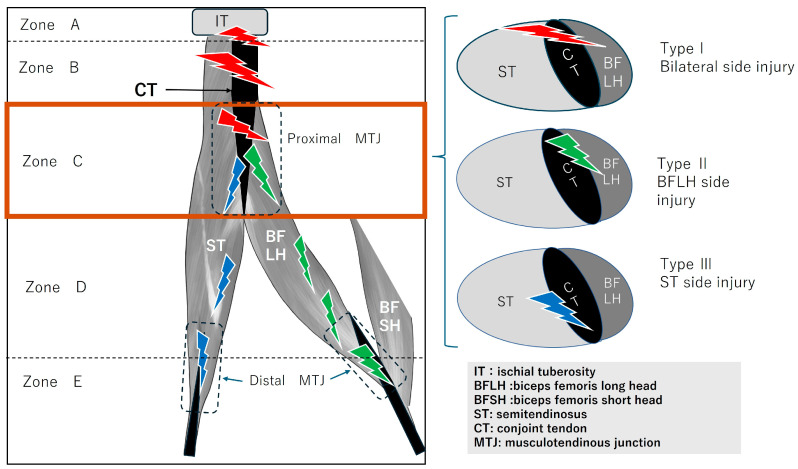
Determination of unilateral or bilateral involvement in Zone C (muscle-tendon transition zone). Zone C (within the brown thick-lined frame), specifically the muscle-tendon junction of the conjoint tendon, was investigated by classifying muscle tears into three types. The horizontal cross-sections for each type are shown on the right. Type I denotes a complete full-thickness rupture of the conjoint tendon, involving damage to bilateralside of the BFLH and ST (red lightning). Type II denotes a rupture affecting only one side of the BFLH (green lightning). Type III denotes a rupture affecting only one side of the ST (blue lightning). Clinically, this is determined using axial MRI images.

Focusing on the conjoint tendon, the following Zone and Type classifications were applied (Figure 2). The conjoint tendon was divided into Zones A–C based on its detachment from the ischium, representing various patterns of musculotendinous junction injury. In Zone C (Figure 3), the injury location was further classified as ST-side, BFLH-side, or involving both.

### 2.4. Definition of RTP Criteria

During the examination, physical findings and imaging diagnostics were conducted. Communication was maintained with the team’s dedicated trainer. Agility tests, mechanical load tests on the hamstrings, and practical tests such as tackling were performed to facilitate return to play. Upon passing all these assessments, the player rejoined general team training. This point was considered the return to play. The period until return to play (RTP) was counted from the day of injury as day 0.

RTP time was compared across the different Types. Among Type 1 proximal conjoint tendon injuries, differences in RTP were analyzed according to injury site and severity. In Zone C injuries, RTP time was compared for each Type.

### 2.5. Statistical Analysis

Statistical analyses were performed using SPSS Statistics (Version 27; IBM Corp., Armonk, NY, USA). Normality of RTP time (weeks) for each group was assessed using the Shapiro–Wilk test, and homogeneity of variance was evaluated using Levene’s test. If equal variances were confirmed, one-way analysis of variance (ANOVA) was used; if not, the Kruskal–Wallis test was applied. Post hoc comparisons were conducted using the Mann–Whitney U test with Bonferroni correction. RTP duration is presented as the mean with 95% confidence intervals (CIs). The 95% CIs were calculated based on the standard error of the mean. Statistical significance was set at *p* < 0.05. The study population represents a convenience sample, including all eligible athletes who presented during the study period and met the predefined inclusion criteria. Owing to the observational and exploratory nature of the study, no a priori sample size calculation was performed.

### 2.6. Ethics Approval

Ethics approval was obtained from the institutional clinical research ethics committee of Waseda University (approval date: 23 September 2020, approval code 2020-224). All participants were informed about the purpose and details of the study prior to enrollment, and written informed consent was obtained from each participant.

## 3. Results

A total of 41 male university rugby players were included in the final analysis. Baseline characteristics of the study participants, including age, playing position, and injury side, are summarized in Table 1. Initially, 68 athletes with acute proximal hamstring injuries were screened. Among these, four players with bruises, during the initial consultation, it was not possible to determine, but during the consultation following the MRI, it was revealed that eight athletes had sustained a hamstring injury in a different location on the same side within the past year, and two players who had undergone ipsilateral hamstring tendon harvest for anterior cruciate ligament (ACL) reconstruction were excluded. MRI examinations were performed within seven days of injury using 3.0-Tesla scanners (Discovery MR750W, GE Healthcare Technologies, Inc., Chicago, IL, USA). Thirteen of the 54 cases involved isolated semimembranosus (SM) injury. After these exclusions, 41 players with proximal injuries involving the biceps femoris–semitendinosus (BF–ST) complex were included in the final cohort. BFSH injuries were not observed. No cases demonstrated combined injury involving both the SM and the BF–ST complex.

The mean age at the time of injury was 20.4 ± 1.3 years (range, 18–23 years). The cohort included 26 backs (63%) and 15 forwards (37%). Most injuries occurred during running or sprinting. The original dataset used to generate Table 1, Table 2 and Table 3 is provided in the Supplementary File S1.

**Table 1:** Baseline characteristics of the study participants.

Variable	Category	Value
Participants		41
Age(years)		20.4 ± 1.3
Playing position	Forwards	15 (37%)
	Backs	26 (63%)
Injury side	Right	23 (56%) *
	Left	18 (44%) *

*: The two players sustained injuries to their left and right sides over a period of more than one year.

### 3.1. Comparison of RTP Time by Type

Type I [Conjoint tendon injury] showed the longest RTP duration (11.4 weeks; 95% CI: 9.83–12.97), compared with Type II [BFLH-only injury] (5.28 weeks; 95% CI: 4.48–6.07) and Type III [ST-only injury] injuries (4.00 weeks; 95% CI: 2.76–5.24). Type I was significantly longer than Type II and Type III. There was no significant difference between Type II and Type III (*p* = 0.45) (Table 2).

**Table 2:** Type-specific RTP period. Abbreviations: CI, confidence interval.

Type	*N*	Mean (Weeks)	95% CI (Weeks)	*p*-Value vs. Type I
I	20	11.4	9.8–13.0	―
II	18	5.3	4.5–6.1	<0.01
III	3	4.0	2.8–5.2	<0.01

### 3.2. Zone C Injury Analysis

In Zone C, which corresponds to the proximal musculotendinous junction, five cases involved injuries extending to both sides of the conjoint tendon. Unilateral BFLH injury was the most common pattern occurring in 10 cases. There were no cases of unilateral ST injury. Bilateral (i.e., full-thickness) CT lesions resulted in a significantly longer period to return to competition compared with CT lesions affecting only the BFLH side (i.e., non-full-thickness) (Table 3).

**Table 3:** Comparison of RTP Times for Injuries in Zone C.

Group	*N*	Mean (Weeks)	95% CI (Weeks)	*p*-Value
Bilateral side injury	5	11.0	7.7–14.3	
BFLH side injury	10	6.1	4.4–7.8	0.042 *
ST side injury	None	―	―	

Notes: Independent samples Mann–Whitney U test * *p* < 0.05. Abbreviations: CI, confidence interval; ST, semitendinosus; BFLH, biceps femoris long head.

### 3.3. Analysis of Injury Location and RTP in Type I

In Type I injuries, RTP duration varied according to injury zone and grade (Table 4). Zone A injury (Grade 2) showed an RTP of 20.0 weeks, based on a single case. In Zone B injuries, RTP increased with higher injury grade, from 7.5 weeks (95% CI: 1.2–13.9) in Grade 1 to 14.4 weeks (95% CI: 4.9–23.9) in Grade 3. Zone C injuries (Grade 2) demonstrated a mean RTP of 11.0 weeks (95% CI: 6.3–15.7).

### 3.4. Case Presentations

#### 3.4.1. Case 1—Type I/Zone A (Conjoint Tendon Detachment), Grade 2

**Figure 4:**
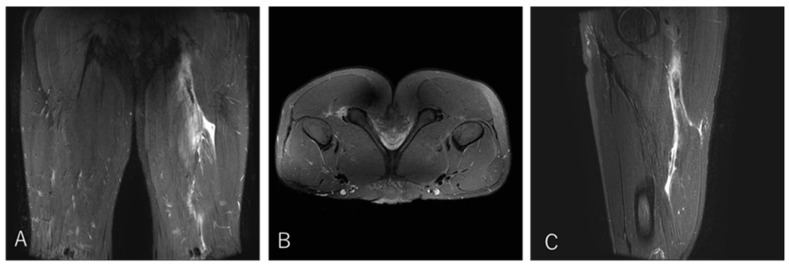
Case 1 MRI images: Conjoint tendon detachment, Type I. (**A**) (Coronal image): The CT (conjoint tendon) is damaged near the ischial tuberosity, with the haematoma extending to the MTJ. However, the retraction of the ruptured tendon is not severe. (**B**) (Axial image): prone position, The CT is detached from the ischial tuberosity in zone A. The SM tendon is normal. (**C**) (Sagittal image): The injury is located proximally to the CT, with the haematoma extending distally into the inter-muscular space.

A 20-year-old lock (FW) stepped to evade an opponent while running with the ball and experienced a sharp tearing sensation in the posterior thigh near the origin. He returned to competition after 20 weeks (Figure 4).

#### 3.4.2. Case 2—Type I/Zone B (Conjoint Tendon Tear), Grade 3

**Figure 5:**
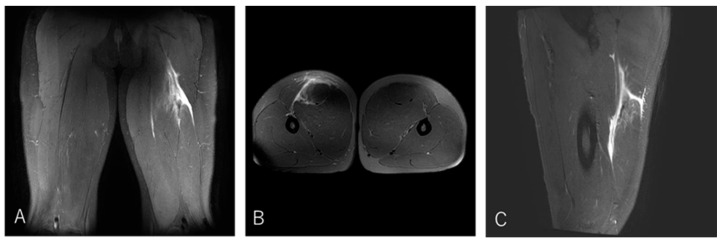
Case 2 MRI images: Conjoint tendon tear, Type I. (**A**) (Coronal image): Complete rupture of the conjoint tendon (CT). A haematoma is present where part of the CT is not visualised. The MTJ has retracted distally. (**B** )(Axial image): prone position, A defect on CT and haemorrhage are observed at the rapture site. (**C**) (Sagittal image): The ruptured distal CT tendon has retracted.

A 20-year-old flanker (FW) experienced sharp twisting pain during a 30 m sprint drill while running at top speed. He returned to play after 14 weeks (Figure 5).

#### 3.4.3. Case 3—Type I/Zone C (MTJ Injury), Grade 2

**Figure 6:**
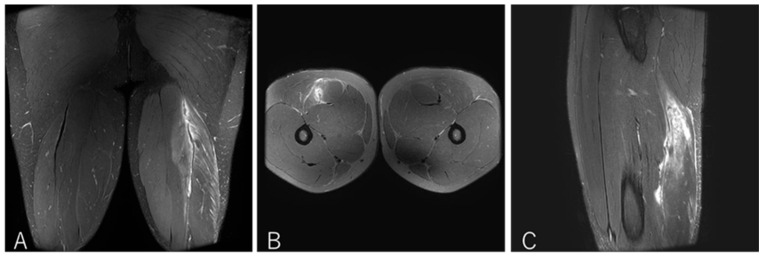
Case 3 MRI images: Conjoint tendon MTJ injury, Type I. (**A**) (Coronal image): The injured area was the proximal MTJ on CT, with haemorrhage observed bilaterally and tortuosity noted on CT. (**B**) (Axial image): prone position, bleeding is present on both BFLH and ST sides of the CT, and the CT is interrupted at the rupture site. (**C**) (Sagittal image): The CT is slightly retracted and accompanied by a haematoma.

A 21-year-old scrum half (BK) developed pain in the proximal posterior thigh while changing direction to chase an opposing player. He returned to play after 10 weeks (Figure 6).

#### 3.4.4. Case 4—Type II/Zone C (Proximal MTJ Tear), Grade 2

**Figure 7:**
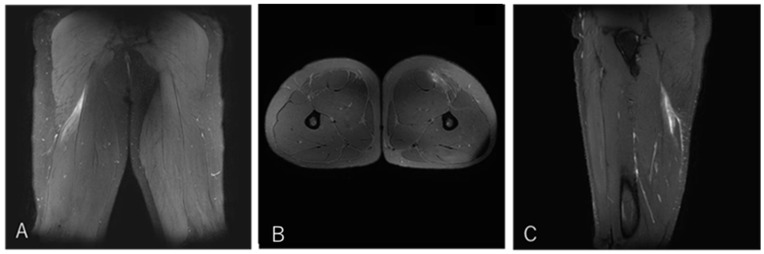
Case 4 MRI images: BFLH proximal MTJ injury, Type II. (**A**) (Coronal image): This is an injury to the proximal musculotendinous junction of the CT, with no evidence of tendon tortuosity. Haemorrhage is present on the BFLH side. (**B**) (Axial image): prone position, A haematoma is noted on the BFLH side of the CT. (**C**) (Sagittal image): A haematoma is observed in the cross-section of the BFLH.

A 20-year-old center (BK) developed posterior thigh pain during a match while tackling an opponent. He returned to play after three weeks (Figure 7).

#### 3.4.5. Case 5—Type II/Zone C (Proximal MTJ Tear), Grade 3

**Figure 8:**
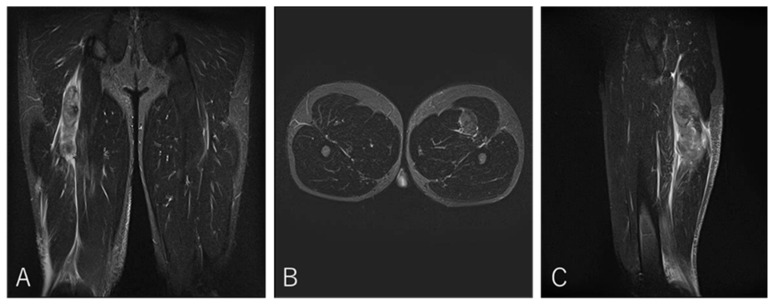
Case 5 MRI images: BFLH proximal MTJ injury, Type II. (**A**) (Coronal image): The tendon is extensively ruptured from the proximal portion of the MTJ with the CT of the BFLH. (**B**) (Axial image): prone position, Extensive haematoma is observed on the BFLH side of the CT. A similar appearance was observed from the proximal to the distal cross-sections. (**C**) (Sagittal image): Extensive haematoma was observed in the cross-section of the BFLH.

A 20-year-old wing (BK) developed sharp pain accompanied by a snapping sensation while accelerating from a low crouching position during a match. He returned to competition after nine weeks (Figure 8).

#### 3.4.6. Case 6—Type III/Zone E (Distal MTJ Tear), Grade 2

**Figure 9:**
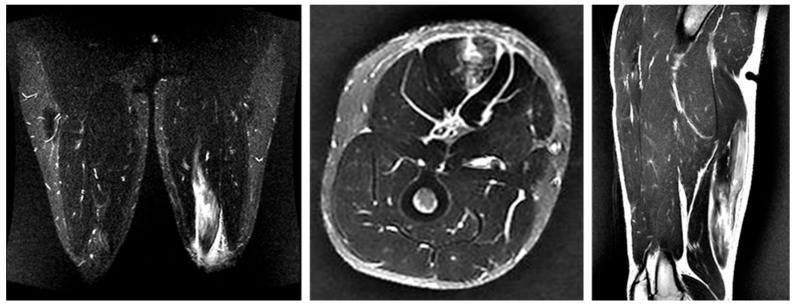
Case 6 MRI images: ST distal MTJ injury, Type III. (**A**) (Coronal image): A haemorrhagic lesion is observed at the distal MTJ. (**B**) (Axial image): prone position, A lesion of the intramuscular tendon of the ST distal tendon is observed. (**C**) (Sagittal image): A lesion is observed at the distal MTJ of the ST.

A 20-year-old center (BK) developed thigh pain while running with the ball during a match. He returned to competition after six weeks (Figure 9).

## 4. Discussion

The present study demonstrates that return-to-play (RTP) duration after proximal hamstring conjoint tendon injury is primarily influenced by injury type, with Type I injuries requiring the longest RTP, whereas Type II and Type III injuries showed substantially shorter recovery times. These findings indicate that injury type reflects fundamental differences in structural involvement within the BF–ST complex and serves as a key determinant of recovery time. Beyond injury type, the present study provides further insight into the clinical relevance of proximal conjoint tendon involvement, particularly in Zone C, which corresponds to the proximal musculotendinous junction. In this zone, injuries extending to both sides of the conjoint tendon (full-thickness CT lesions) resulted in a significantly longer RTP than injuries confined to the BFLH side (non-full-thickness CT lesions), as shown in Table 3. These finding highlights that, within the BF–ST complex, the extent of conjoint tendon involvement—rather than anatomical location alone—plays a critical role in delayed recovery. Collectively, these results underscore that proximal conjoint tendon injury has a substantial impact on RTP by altering load sharing and functional integrity within the BF–ST complex, thereby supporting the clinical importance of evaluating both injury type and full-thickness involvement of the conjoint tendon when estimating prognosis.

### 4.1. Anatomical and Biomechanical Perspectives of the BF–ST Complex

The conjoint tendon (CT) is a proximal tendinous structure shared at the ischial tuberosity by the lateral hamstring, the long head of the biceps femoris (BFLH), and the medial hamstring, the semitendinosus (ST) [16,17,33,34,35]. In addition, the semimembranosus (SM) attaches independently in the same region [35,36,37,38,39,40]. Previous electromyographic and biomechanical investigations have shown that the functional contribution of individual hamstring muscles is not uniform but varies according to sprint phase and mechanical demand. Rather than acting synchronously, the biceps femoris long head and semitendinosus demonstrate phase-dependent roles, resulting in heterogeneous loading patterns within the proximal conjoint tendon. Such non-uniform activation may increase localized mechanical stress during high-intensity movements, particularly at phases requiring rapid force transmission, thereby predisposing the conjoint tendon to delayed healing when injured [21,41]. Ono reported, based on electromyography and muscle functional MRI, that hip extension relies more on the BF and SM than on the ST [23]. During the terminal phase of sprinting and change-of-direction movements (late swing to early ground contact), BFLH generates an external rotation moment, whereas ST and SM generate internal rotation moments, creating opposing tensile forces concentrated in the central portion of the CT [22,42]. These opposing forces may destabilize tendon fiber realignment during the healing process, increasing the risk of scar formation and re-rupture. These findings support our hypothesis that the CT, subjected to opposing traction forces from its medial (ST) and lateral (BFLH) components, contributes to delayed healing and prolonged time to return to competition.

### 4.2. Zone C (Proximal MTJ Injury): Unilateral or Bilateral?

We investigated the frequency and recovery time of three injury patterns in Zone C, the musculotendinous junction: unilateral BFLH injury, unilateral ST injury, and bilateral injury involving both the BFLH and ST. In this study, there were no cases of unilateral ST injury in Zone C, and bilateral injuries resulted in longer recovery times than unilateral BFLH injuries. Pollock et al. (2014) classified hamstring strain morphology into myofascial injuries, myotendinous injuries, and intratendinous injuries of the BFLH [32]. This supports the notion that Type I injuries, being intratendinous in nature, require a longer recovery period. He reported that intratendinous injuries take the longest to heal because of poor blood supply and the characteristics of the extracellular matrix [36]. This suggests that unilateral injuries correspond to myotendinous injuries, whereas bilateral injuries correspond to intratendinous injuries. In other words, bilateral injuries at the musculotendinous junction share the structural characteristic that tendon healing is prolonged [4,43,44]. Balius et al. [45,46] explained delayed tendon repair in terms of impaired ECM remodeling: the ECM provides mechanical strength and elasticity to tendon tissue, and after injury it requires a long time to synthesize and reorganize collagen types I and III. In particular, the ECM in tendons is hypometabolic, and the rate of tendon repair is significantly slower than that of muscle fiber injury, leading to delayed RTP [47,48].

### 4.3. Location of Conjoint Tendon Injury and Return to Play

How much difference in recovery time should be expected depending on the specific site of injury within the proximal conjoint tendon, which serves as the shared origin for the medial (ST) and lateral (BFLH) hamstrings? Although Zone A included only one case, the Grade 2 injury in this zone required 20 weeks for RTP. This recovery time was clearly longer than that of injuries in Zones B and C. In other words, injuries at the insertion of the conjoint tendon took significantly longer to recover than injuries to the tendon itself. No clear difference was observed between Zones B and C. This study found that even for musculotendinous junction injuries, when both the ST and BFLH were involved, the recovery time was similar to that of Zone B injuries, which involve the free tendon of the BFLH (Table 3). Askling (2007) [12] reported that BFLH injuries closer to the ischial tuberosity required longer recovery times. Considering Zone C injuries as those involving either the BFLH alone or both the BFLH and ST (*N* = 15), the mean RTP was 7.7 weeks, showing a pattern of Zone A > Zone B > Zone C, consistent with the findings of the present study.

### 4.4. Injury Mechanism and Player’s Position

The injury mechanism in this study was predominantly muscle strain occurring during running, with few stretch-type injuries involving the hamstrings, which are more likely to occur during actions such as mauls. Regarding player position, injuries were more common among backs (positions 9–15) than forwards (positions 1–8). These findings are consistent with previous reports by Askling [12,49]. These anatomical and physiological factors, together with previous findings, support the conclusion that RTP following conjoint tendon injuries is prolonged.

### 4.5. Comparison with Previous Classifications and Novelty

Numerous classifications for hamstring injuries have been proposed. In the Munich Consensus by Müller-Wohlfahrt et al. (2013), injury morphology was divided into functional and structural injuries, with structural injuries further classified in detail by site and extent [30]. The BAMIC classification by Pollock et al. (2014) categorizes injury sites into myofascial, myotendinous, and intratendinous types, and assigns a severity grade from 0 to 4 [32]. Although these existing classifications have been useful and have contributed to the standardization of injury location and severity, none specifically distinguishes injuries of the conjoint tendon (CT), which is shared by the BFLH and ST.

To our knowledge, this is the first prospective study to identify full-layer CT injuries as a distinct entity and to quantitatively demonstrate their significant impact on RTP in competitive athletes. In this study, we focused on the ST–BF complex, which shares a proximal conjoint tendon, and classified injuries into three categories: Type I, full-layer conjoint tendon tear involving both the BFLH and ST; Type II, BFLH-only injury; and Type III, ST-only injury. The key novelty of this classification is that it enables clear assessment of the presence or absence of conjoint tendon injury and provides clinically relevant information directly related to RTP prediction. In particular, this study differs from previous classifications in demonstrating that Type I injuries have a significantly longer RTP than the other injury types.

### 4.6. Confounders and Limitations

Several potential confounding factors should be considered when interpreting the present findings [50]. Although a standardized rehabilitation framework was applied within the team, individual variability in rehabilitation progression, adherence, and tolerance to loading may have influenced return-to-play (RTP) duration. In addition, playing position represents a potential confounder, as physical demands, exposure to contact, and return-to-play criteria differ between forwards and backs. Importantly, players who had previously suffered a muscle strain in the same area or who had an injury in the different area in the same side and had returned within one year were excluded from this study to minimize the influence of residual deficits or reinjury-related factors on RTP. However, while major prior hamstring surgery was excluded, the potential impact of remote or contralateral hamstring injury history could not be completely controlled for and may still have influenced recovery.

This study also has several important limitations. First, the study was conducted in a single cohort of male university rugby players, and the overall sample size was relatively small, particularly for Type III injuries, limiting the statistical power of between-group comparisons and the external validity of the findings. Caution is therefore required when generalizing these results to athletes from other sports, competitive levels, or female populations. Second, all MRI examinations were performed using a 3.0-Tesla scanner, ensuring consistency in image acquisition. Nevertheless, MRI was obtained within seven days of injury, and variability in imaging timing may have affected the assessment of edema, hemorrhage, or early tendon discontinuity. Third, classification of injury Type, Zone, and Grade was performed by experienced physicians; however, inter-rater reliability was not formally assessed, which may have introduced classification bias. Finally, RTP was defined as full return to team training or match play, an outcome that may be influenced by team circumstances, coaching decisions, and playing position, rather than reflecting purely biological tissue healing. Moreover, analyses involving small subgroups, particularly zone- and grade-based categories, should be interpreted cautiously and are best regarded as exploratory, intended to generate hypotheses rather than establish definitive conclusions.

### 4.7. Clinical Implications

The findings of the present study have several important clinical implications for prognosis estimation, return-to-play (RTP) planning, and rehabilitation management following proximal hamstring conjoint tendon injury. First, the clear differences in RTP duration according to injury type indicate that early classification of injury morphology can provide clinicians with a realistic framework for initial prognosis and communication with athletes and coaching staff. Second, the results from Zone C injuries suggest that assessment of conjoint tendon involvement, particularly whether the lesion is full-thickness or non-full-thickness, may be useful for anticipating a prolonged recovery course. Recognition of full-thickness conjoint tendon injury at the proximal musculotendinous junction may therefore prompt more cautious RTP timelines and closer monitoring during rehabilitation. Finally, these findings support a graded rehabilitation strategy, in which rehabilitation intensity and progression are tailored according to injury type and the extent of conjoint tendon involvement. Athletes with more extensive conjoint tendon injury may benefit from a slower progression of loading and sport-specific activities, whereas those with less extensive involvement may tolerate earlier advancement. Collectively, integrating injury type, zone, and tendon involvement into clinical decision-making may improve RTP planning and help balance the competing demands of timely return and injury recurrence prevention.

## 5. Conclusions

Full-layer proximal hamstring conjoint tendon tears, particularly bilateral Zone C injuries, are associated with an approximately twofold longer return-to-play (RTP) duration compared with non-full-layer injuries. MRI-based classification of injury type, zone, and extent of conjoint tendon involvement should be used to guide prognosis, return-to-play planning, and rehabilitation intensity.

## Figures and Tables

**Table 4:** Duration of RTP by Zone and Grade for Type I Injuries.

Zone	Grade	*N*	Mean (Weeks)	95% CI (Weeks)
A	2	1	20.0	-
B	1	2	7.5	1.2–13.9
B	2	7	9.4	7.3–11.6
B	3	5	14.4	4.9–23.9
C	2	5	11.0	6.3–15.7

## Data Availability

The data presented in this study are available on request from the corresponding author (M.W.). The data are not publicly available due to privacy concerns.

## Supplementary Materials

The following supporting information can be downloaded at: https://www.mdpi.com/article/10.3390/diagnostics16010166/s1, Supplementary File S1: data of subjects: Original anonymized dataset of all subjects used to generate Table 1, Table 2 and Table 3, including demographic variables, injury characteristics, and return-to-play time.
